# Tumor SOCS3 methylation status predicts the treatment response to TACE and prognosis in HCC patients

**DOI:** 10.18632/oncotarget.16157

**Published:** 2017-03-13

**Authors:** Bei-Ge Jiang, Neng Wang, Jian Huang, Yuan Yang, Liang-Liang Sun, Ze-Ya Pan, Wei-Ping Zhou

**Affiliations:** ^1^ Department of Surgery, Eastern Hepatobiliary Surgery Hospital, Second Military Medical University, Shanghai, P.R. China; ^2^ Department of Endocrinology, Shanghai Changzheng Hospital, Second Military Medical University, Shanghai, P.R. China

**Keywords:** suppressor of cytokine signaling, transarterial chemoembolization, methylation, prognosis

## Abstract

**Background:**

Suppressor of cytokine signaling (SOCS) 1 and 3 methylation have been associated with clinical features and outcomes of cancer patients. However, their roles in determining the treatment response to transarterial chemoembolization (TACE) in patients with hepatocellular carcinoma (HCC) remain unknown.

**Results:**

We found that presence of SOCS3 methylation is significantly associated with the major clinical features of HCC patients, including tumor stage, lymph node and vascular invasion. Of note, we observed that the presence of SOCS3 methylation is closely related to TACE response. In prognosis analyses, HCC patients with SOCS3 methylation presence have a poorer prognosis indicated by lower 3-, and 5-year survival rates and shorter mean survival period, than those without. Multivariate COX analysis confirms the prognostic role of the presence of SOCS3 methylation in HCC patients receiving TACE treatment.

**Materials and Methods:**

A total of 246 HCC patients receiving TACE were enrolled in this study. Tumor samples was obtained from echo-guided fine needle aspiration and genomic DNA from tumor samples was purified. SOCS1 and SOCS3 methylation status were detected using methylation-specific polymerase chain reaction. The treatment responses to TACE of patients were evaluated after procedure and all patients were followed for prognosis analysis.

**Conclusions:**

This finding suggests that the presence of SOCS3 methylation is a marker to predict treatment response and prognosis in HCC patients receiving TACE therapy.

## INTRODUCTION

Hepatocellular carcinoma (HCC) is one of the most frequent cancers worldwide. Despite of the recent progress in diagnosis and treatment, the clinical outcome of HCC patients remains very poor [1–3]. Many patients lose timing for tumor resection due to late diagnosis. Transarterial chemoembolization (TACE) is a new technique of intra-arterial catheter-based chemotherapy that selectively delivers cytotoxic drug to the tumor bed combining with arterial embolization. For these patients not eligible for surgical treatment, TACE is currently considered as part of standard therapy [4, 5]. However, treatment response to TACE in individual patient varies widely. Although many efforts have been made, there are still no reliable markers to predict treatment response to TACE and prognosis in HCC patients.

Recent studies show that DNA methylation is one of major molecular mechanisms in carcinogenesis in liver. Increasing evidence reveals that HCC tumors exhibit specific DNA methylation signatures that are associated with major risk factors and tumor progression. Using methylation-specific PCR (MSP), some genes are identified significantly hypermethylated and some are downregulated in the HCC tumors compared to the non-tumor liver tissues [6]. Other studies show that the methylation status of some candidate genes is closely associated with HCC progression and prognosis [7–10].

Suppressor of cytokine signaling (SOCS) family is an important negative regulator of cytokine signaling and deregulation of SOCS has been involved in many types of cancer. Among SOCS family members (SOCS1-7), the role of SOCS1 and SOCS3 in are mostly studied. The roles of SOCS1 and SOCS3 in various types of cancer are still controversial. Some researchers found there increased expression of these two in tumor samples while the others reported reduced expressions, which make it hard to use the protein expression of these two for tumor maker [11–13].

SOCS3 gene has been reported to be hypermethylated in various types of cancers, including endometrial carcinoma, prostate cancer, Barrett esophagus carcinoma, and ulcerative colitis-related colorectal cancer [14–16]. The *in vitro* study reveals that SOCS3 methylation promotes cell growth in pancreatic cancer cell line [17]. Aberrant promoter methylation and loss of suppressor of SOCS1 gene expression was reported in uterine cervical carcinogenesis [18]. SOCS-1 hypermethylation is significantly correlated with lymph node metastasis and TNM stage in colorectal cancer [19]. The frequency of SOCS-1 methylation in HCC cancer tissues is significantly higher than in adjacent non-tumorous tissues and benign liver tissues, but no prognostic effect of SOCS-1 methylation was observed in HCC patients [20].

Identification of potential biomarker that can predict the treatment response after procedure is important to improve the survival of HCC patients receiving TACE treatment. The aim of this study was to test whether SOCS1 and 3 methylation status are associated with the treatment response HCC patients receiving TACE. We found a close relation between SOCS3 methylation status and TACE treatment response, as well as survival of HCC patients, suggesting SOCS3 methylation status can be used as a marker to predict the TACE treatment response and prognosis of HCC patients.

## RESULTS

### SOCS1 and 3 methylation status and the clinical features and treatment response of HCC patients

The tumor samples from patient were analyzed for SOCS1 and 3 methylation status by MSP assay. SOCS1 methylation was identified in 105 (42.7%) of the tumor tissues and the number for SOCS3 methylation status are164 (66.7%). We then analyzed the association between SOCS1 and 3 methylation status and the clinical features. We found that the SOCS3 methylation was significantly associated with following clinical features including Child-Pugh classification, TNM stage, lymph node invasion, vascular invasion and serum AFP level (Table 2). As for SOCS1 methylation status, we found that it is related to vascular invasion and TNM stage (Table 1).

**Table 2:** Identification of prognostic factors for overall survival in patients with TACE treatment

	Univariate analysis			Multivariate analysis		
	HR	95% CI	*P*	HR	95% CI	*P*
Lymph node invasion	1.43	1.02–2.56	0.026	1.23	0.89–2.34	0.058
Vascular invasion	2.56	1.67–4.56	0.013	2.33	1.45–4.22	0.014
TNM (III–IV vs. I–II)	2.13	1.23–3.98	0.024	1.99	1.15–3.45	0.028
AFP (> 200 vs. < 200)	1.28	1.04–3.05	0.034	1.17	0.78–3.24	0.054
TACE response (Poor vs. Well)	2.75	2.05–5.37	< 0.001	1.76	2.02–5.32	0.001
SOCS3 Methylation vs.Unmethylation	3.56	2.67–6.56	< 0.001	3.44	2.57–6.31	< 0.001

HR, Hazard Ratio; CI, Confidence interval.

**Table 1:** SOCS1 and 3 methylation status and the clinical features and treatment response of HCC patients

	Methylation	SOCS1	*P*	Methylation	SOCS3	*P*
		Unmethylation			Unmethylation	
**AGE**						
> 50	50	76	0.453	90	46	0.483
< 50	55	65		74	36	
**Sex**						
Male	71	97	0.476	110	58	0.333
Female	34	44		54	24	
**Etiology**						
HBV	93	128	0.359	147	74	0.538
HCV	12	13		17	8	
**Child-Pugh classification**						
A	81	109		115	75	0.001
B	24	32		49	7	
**Lymph node invasion**						0.024
Presence	17	17	0.288	28	6	
Absence	88	124		135	76	
**Vascular invasion**						0.002
Presence	38	19	0.001	47	10	
Absence	67	122		117	72	
**TNM**						0.001
I–II	40	84	0.006	114	58	
III–IV	66	57		50	24	
**Tumor number**						
Single	69	103	0.136	81	41	0.483
Multiple	36	38		83	41	
**Size**						
< 5	59	69		84	44	0.411
> 5	46	72		80	38	
**AFP**						
< 200	52	76	0.496	41	67	0.001
> 200	53	65		123	15	
**TACE response**						
Poor	72	23	0.011	43	52	0.06
Well	92	59		85	66	

We next anal size the association of SOCS1 and 3 methylation status with the treatment response to TACE in studied cohorts. Of note, we observed that

There are 72 (43.9%) patients had poor response to TACE in SOCS3 methylation group, while in unmethylation group, only 23 patients (28.1%) responded poorly to TACE (*P* = 0.011, Table 1). In contrast, SOCS1 methylation status does not affect the TACE response status in this study (*p* = 0.06, Table 1).

### SOCS3 methylation status and prognosis of HCC patients

**Figure 1:**
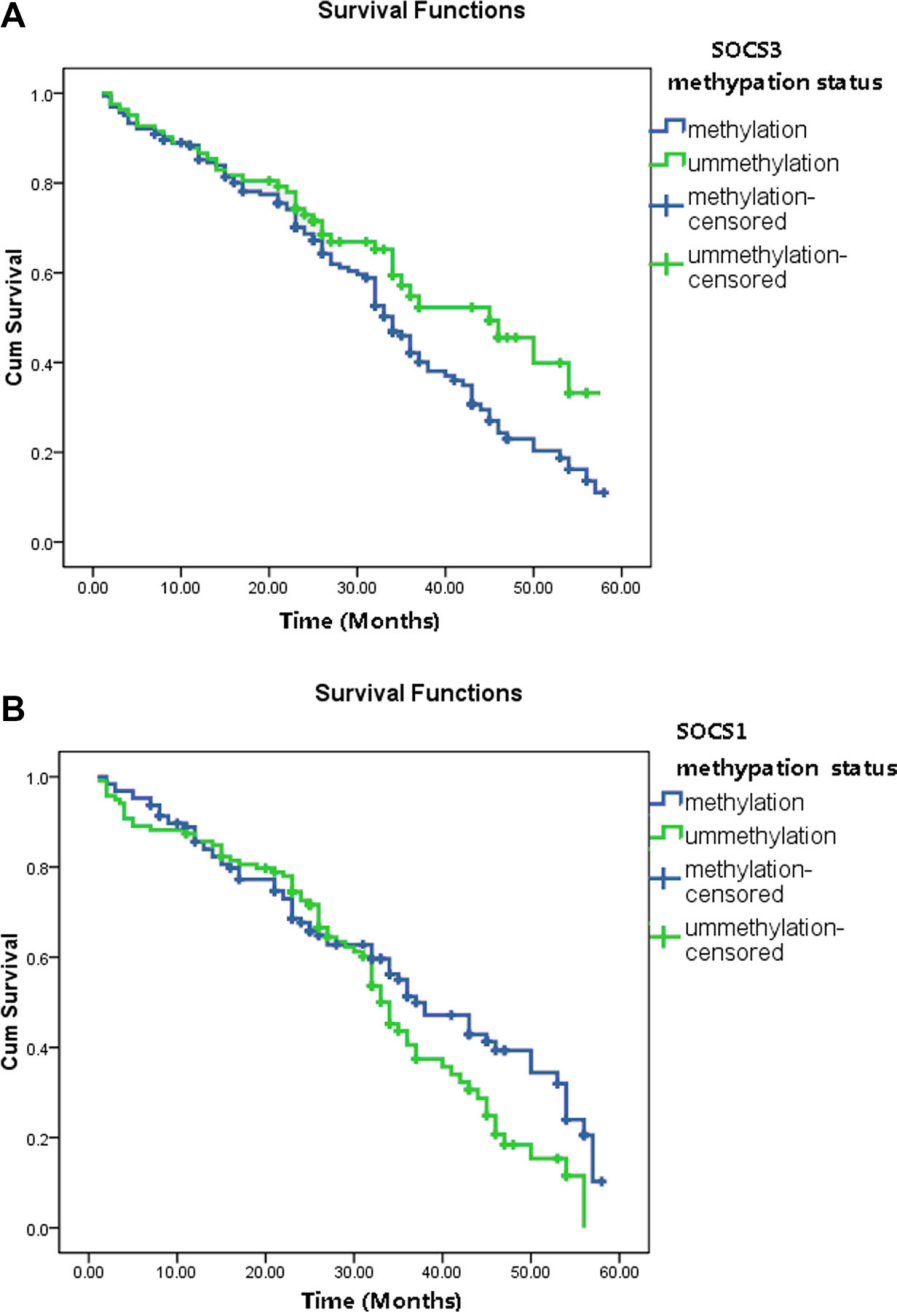
The overall survival (OS) status in HCC patients based on tumor SOCS1 and SOCS3 methylation status (**A**) The 3-year and 5-year survival rates are significantly different between patients with SOCS3 methylation and unmethylation (54.6% vs 38.5%, *P* < 0.001 and 36.5% vs.11.6%, *P* < 0.001, respectively). HCC patients with SOCS3 methylation have a poorer prognosis than those without (median OS period: 22.5 vs. 29.7, months, *P* < 0.001, log-rank test). (**B**) The 1-year, 3-year and 5-year survival rates as well as the median OS period are not significantly different between patients with SOCS1 methylation and unmethylation (all *P* > 0.05).

To determine whether SOCS1 and SOCS3 have a prognostic role in these patients after TACE treatment, we analyzed the overall survival (OS) status using Kaplan-Meier model and the 1-, 3-, and 5-year survival rates were calculated. Our results show that, the 1-year survival rates are similar between patients with/without SOCS3 methylaiton presence. However, the 3-year and 5-year survival rates are significantly different between these two groups (54.6% vs 38.5%, *P* < 0.001 and 36.5% vs.11.6%, *P* < 0.001, respectively). The median survival times of two groups are also dramatically different. HCC patients with SOCS3 methylation have a poorer prognosis than those without (median OS period: 22.5 vs. 29.7, months, *P* < 0.001, log-rank test, Figure 1A). However, no significant correlation was observed between SOCS1 methylation status and OS in HCC patients (Figure 1B).

Furthermore, univariate and multivariate Cox analysis were performed to identify the prognostic factors for overall survival in patients with TACE treatment. As shown in Table 2, TNM stage, lymph node invasion, vascular invasion, serum AFP level, SOCS3 methylation as well as TACE response were screened as possible factors associated with the survival of HCC patients. Notably, multivariate analysis reveals that SOCS3 methylation status and TACE response, along with TNM stage and Vascular invasion, are identified as independent risk predictors for the poor outcome of HCC.

## DISCUSSION

In this study, we investigated the potential relation of the methylation status of *SOCS1 and SOCS3* with the treatment response to TACE and post-procedure outcome in HCC patients. We found that *SOCS3* methylation status is closely associated with TACE response and prognosis in the studied cohorts, suggesting SOCS3 methylation status may be used as a biomarker for predicting the therapeutic effect of TACE in HCC patients.

DNA methylation is the addition of a methyl group to the cytosine in CpG dinucleotides by DNA methyltransferase. This epigenetic mechanism is used by cells to regulate gene expression. Hypermethylation associated with silencing of SOCS proteins has been found in various cancers, including myeloma, melanomas, bladder, HCC, gastric and colorectal cancers [25–29]. Pierconti et al. found that the promoter of SOCS3 was methylated in 20 out of 51 (39.2%) prostate cancer patients, whereas all healthy controls and benign prostate hyperplasias were not methylated. SOCS3 methylation decreased mRNA level and significantly associated with a more aggressive behavior and worse prognosis in prostate cancer [30]. Similarly, in multiple myeloma patients, methylation of SOCS3 was found in 5 of the 70 cases but none in the control group [25]. Moreover, association of SOCS3 methylation with plasma cell leukemia, elevated LDH, and shortened survival (6.9 versus 56.1 months) was observed.

SOCS3 is known to inhibit cytokine signaling via Janus kinase (JAK)/signal transducers, activators of transcription [31], NFkB, and focal adhesion kinase (FAK) signaling pathways. Substantial data demonstrate the link between SOCS3 regulation of inflammation and its suppressor activity on tumor initiation and development [11, 32, 33]. In SOCS3 conditional knockout mice, deletion of the SOCS3 gene promoted carcinogen-induced hepatic tumor development through the activation of STAT3 and resistance to apoptosis [34]. In HCC patient samples, the expression of SOCS3 was reduced compared with surrounded non-HCC regions [34], and methylation silencing of SOCS3 accelerates cell growth and cell motility by promoting JAK/STAT and FAK signaling [35]. In line with these findings, our results showed that SOCS3 mRNA level in HCC tissues were significantly lower than adjacent non-tumor tissue. 65.8% of the tumor tissues showed hypermethylation, which was associated with tumor grade, TNM stage and distance metastasis, higher level of AFP, and poorer treatment response. Methylation silencing of the SOCS3 in HCC may result in survival cancer cells via upregulation of cytokine signaling pathways and resistance to apoptosis, which in turn lead to unfavorable response to TACE treatment.

More recently, hypermethylation of SOCS3 was shown to associate with a poor clinical outcome in HCC patients with HBV infection backgrounds but not that with HCV or no virus infection [36]. Interestingly, methylation was also observed in non-tumor tissues in HBV-related HCC patients even though with much lower intensity and frequency. However, no methylation was seen in non-tumor tissues of HCV infection-related HCC, indicating that methylation status of SOCS3 varies under different hepatitis viruses-induced HCC. While our study provided important information on the potential role of SOCS3 methylation on prognosis of TACE, subgrouping based on gender and/or virus infection background of HCC in the future study would be helpful to further our understanding on the mechanism underlying effects SOCS3 methylation in HCC and TACE treatment. Additionally, future study using larger sample size should be performed to further confirm our results.

Another member of the SOCS family, SOCS-1, displays hypermethylation and growth suppression activity through JAK/STAT pathway in HCC as well [37]. According to a meta-analysis, hypermethylation of SOCS1 was correlated to the risk of HCC [38]. In this study, we found SOCS1 methylation are correlated with tumor grade and TNM stage. However, no significant association was observed between SOCS1 methylation status and OS of HCC patients who received TACE treatment, suggesting a unique role of SOCS3 in SOCS protein family in the response to TACE.

Two major limitations should be addressed in this study. Firstly, we did not investigate the biological effect of SOCS3 methylation on HCC cell lines, thus the molecular mechanism underlying SOCS3 methylation affect HCC cell behavior, such as proliferation, migration, invasion, apoptosis et al, remains unknown. Secondly, the sample is relative small and only Chinese patients were enrolled in this study. In order to verify the conclusion of this study, future study with larger sample size and different ethical background is warranted.

In conclusion, our data showed demonstrated a strong correlation between SOCS3 methylation status and the survival of HCC patients received TACE. These results suggest that SOCS3 methylation status could be used for prognosis of TACE in HCC patients, facilitating the clinical application of this new technique.

## MATERIALS AND METHODS

### Patient enrollment

We consecutively enrolled 246 patients receiving TACE procedure due to unresectable HCC at our hospital from May 2010 to May 2014. All patients were diagnosed with HCC by clinical examination, imageology and alpha-fetoprotein (AFP) examination, and further histologically confirmed by echo-guided fine needle aspiration biopsy. Patient's information is collected from their medical records. The characteristics of the HCC patients are presented in Table 1. A written consent was obtained from all patients before enrollment in the study, and the Ethical Committee of our hospital approved the protocol, which was in accordance with the ethical guidelines of the 1975 Declaration of Helsinki.

### TACE procedure and treatment response evaluation

All patients received TACE therapy by the Seldinger technique as described previously. Chemotherapy including Fluorouracil,, Mitomycin, Carboplatin et al, was implemented via super-selective cannulation to the target artery, injection of iodized oil mixture, and gelatin sponge embolism if necessary. All patients received a median of two treatments (range, 1–6 treatments) throughout the follow-up period [21].

The Response Evaluation Criteria in Solid Tumors (RECIST) was used to measure tumor response: CR (complete response), disappearance of all target lesions; PR (partial response), at least a 30% decrease in the sum of the longest diameter of the target lesions; SD (stable disease), neither PR nor progressive disease; PD (progressive disease), at least a 20% increase in the sum of the longest diameter of the target lesions, or the appearance of new lesions or metastasis [22]. Overall survival (OS) was defined as the period from the date of TACE to death or last follow-up. The end date of the follow-up was May 2014, with a median of 28.9 months (range, 2 months–60 months).

### DNA extraction and methylation assay

We isolated genomic DNA from HCC tumor samples using DNeasy Blood & Tissue Kit (Qiagen, Germantown, MD, USA), followed by treatment with sodium bisulphite, which converts all unmethylated cytosines (C) to uracil (U). To analyze the methylation status, methylation-specific PCR (MSP) was performed using primer pairs specific for methylated and unmethylated DNA, respectively [23]. MSP distinguishes methylated from unmethylated alleles in SOCS1 or SOCS3 based on sequence changes (C to U) induced by sodium bisulfite. MSP-specific primers for SOCS1 [24] and SOCS3 [25] are listed in Table 3.

**Table 3:** The primers for methylation-specific polymerase chain reaction (MSP)

Gene	Primer (forward)	Primer (reverse)	
SOCS3	GGAGATTTTAGGTTTTCGGAATATTTC	CCCCCGAAACTACCTAAACGCCG	Methylation specific
	GTTGGAGATTTTAGGTTTTTGGAATATTTT	AAACCCCCAAAACTACCTAAACACCA	Unmethylation specific
SOCS1	GTTGTAGGATGGGGTCGC GGT CGC	CTACTAACCAAACTAAAATCCACA	Methylation specific
	GTTGTAGGATGGGGTTGT GGTTGT	CTACTAACCAAACTA AAATCCACA	Unmethylation specific

### Statistical analysis

The χ^2^ test or Fisher's exact test were adopted to determine the association between the *SOCS1 and 3* methylation status and the clinicopathological features and TACE treatment response in HCC patients. The Kaplan–Meier analysis with log-rank test was used to analyze HCC prognosis and survival time. COX analysis using univariate and multivariate modes was used to determine the independent prognostic factor for GC patients. All of the statistical analyses were performed by GRAPHPAD PRISM software (GraphPad Software, Inc., San Diego, CA, USA) and SPSS (16.0). In all cases, a *p*-value of less than 0.05 was considered significant.
